# PEComa: A Perivascular Epithelioid Cell Tumor in the Liver—A Case Report and Review of the Literature

**DOI:** 10.1155/2013/904126

**Published:** 2013-12-29

**Authors:** Faseeh Khaja, Allison Carilli, Said Baidas, Aravindhan Sriharan, Shanedelle Norford

**Affiliations:** ^1^Division of Hematology/Oncology, MD Anderson Cancer Center Orlando, 1400 South Orange, MP 700, Orlando, FL 32806, USA; ^2^Pathology Department, Orlando Health, 1414 Kuhl Avenue, Orlando, FL 32806, USA

## Abstract

Perivascular epithelioid cell tumors are soft tissue tumors that can occur in various locations in the body whose incidence is rising. Hepatic PEComas are quite rare and diagnosis involves positivity of Melan-A and HMB45 on immunohistochemistry. Usual treatment is surgery for benign tumors and chemotherapy including mTOR inhibitors for malignant tumors. Here we discuss the radiological and pathological diagnosis, evaluation, and management of a hepatic PEComa. We describe a 51-year-old patient who was diagnosed incidentally after unusual physical exam findings.

## 1. Introduction

The term PEComa was introduced by Zamboni four years after a tumor of the perivascular epitheliod cell was first described by Selvaggi et al. in 2011 [1]. In 2002 the World Health Organization classified PEComa as a mesenchymal tumor composed of histologically and immunohistochemically distinctive perivascular epitheliod cells [2]. The etiology of PEComas remains uncertain. They more commonly affect young female patients and span a wide variety of tumors including angiomyolipoma (AML), clear-cell “sugar” tumor of the lung, lymphangioleiomyomatosis (LAM), clear-cell myomelanocytic tumor of the falciform ligament/ligamentum teres, and other rare clear-cell tumors. The most common primary sites of PEComa at presentation are the uterus, vulva, rectum, heart, breast, urinary bladder, abdominal wall, pancreas, retroperitoneum, and liver [3, 4]. Although PEComas are commonly asymptomatic, they may present with vague pain.

## 2. Case Report

**Figure 1:**
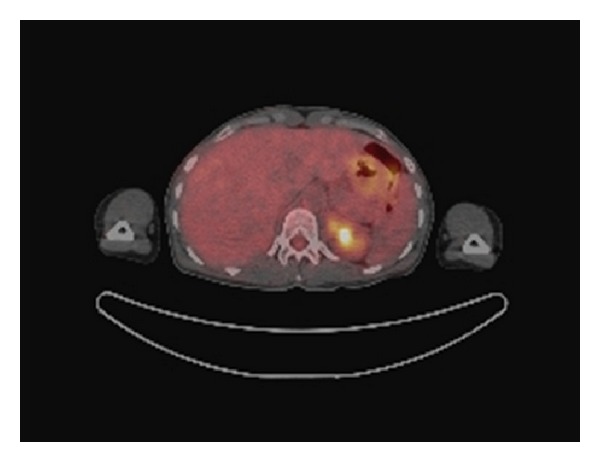
Non-FDG avid liver lesion on PET/CT.

**Figure 2:**
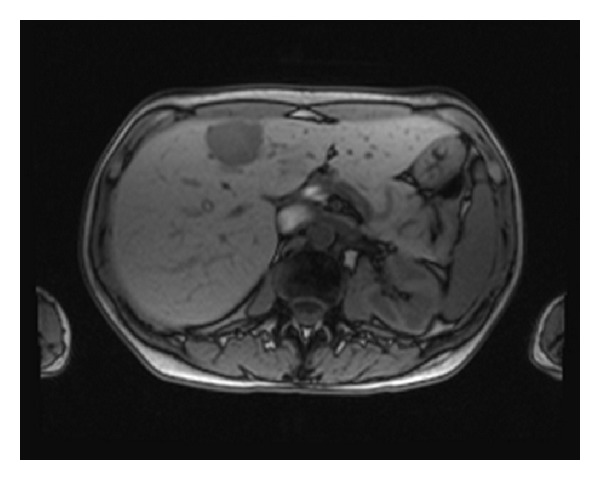
T1 hypointense liver lesion on MRI.

**Figure 3:**
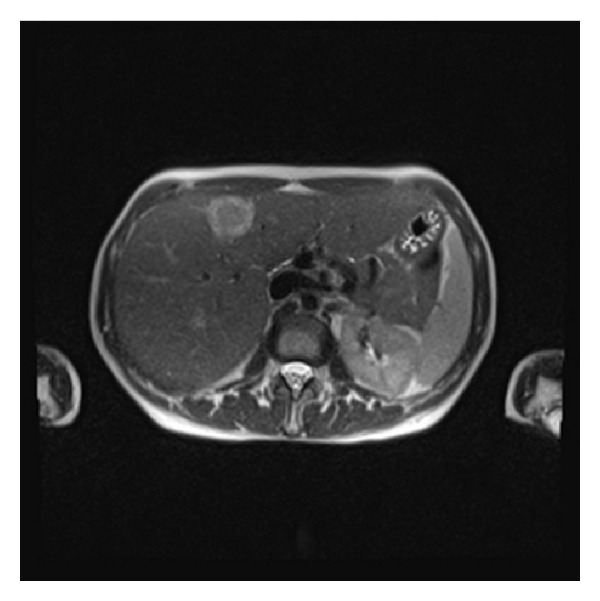
T2 hyperintense liver lesion on MRI.

**Figure 4:**
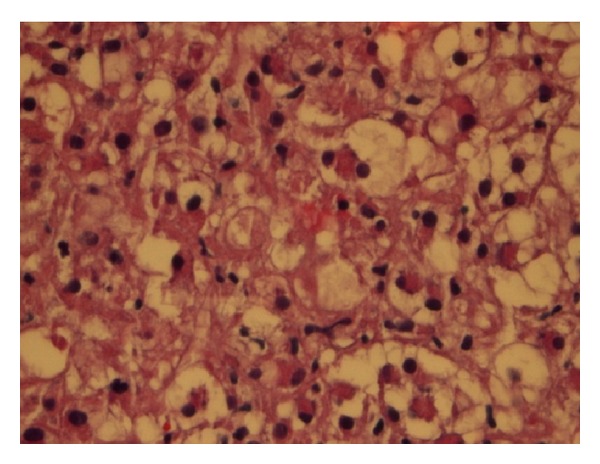
Haphazard arrangement of epithelioid cells with cleared-out cytoplasm.

**Figure 5:**
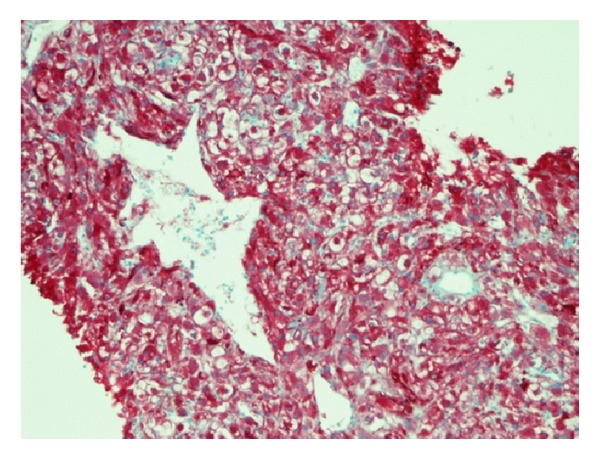
Melan-A stain.

**Figure 6:**
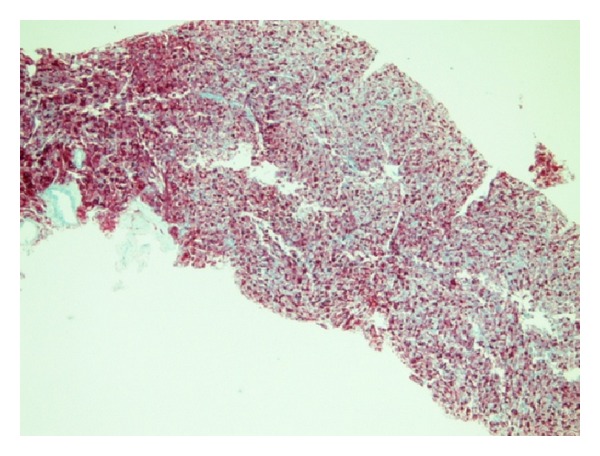
HMB-45 stain.

51-year-old Caucasian female who was previously healthy presented with complaints of skin thickening of her breasts. MRI and mammogram of both breasts were unrevealing. The patient underwent bilateral random breast skin biopsies. Pathology returned as subacute spongiotic dermatitis. She also complained of tender bony prominences in her scalp and hands. On plain X-rays, these were found to be bone islands. Patient worked as a nurse, and due to vague generalized symptoms she insisted on PET scan to be done which revealed a nonmetabolically active lesion in the liver, slightly less metabolically active than the surrounding liver parenchyma, with no other lesions being identified on PET scan as a primary site (Figure 1). A dedicated liver MRI revealed a hypervascular lesion with irregular lobulated margins in the anterior right lobe of the liver near the falciform ligament that was T1 hypotense (Figure 2) and T2 hypertense (Figure 3) with heterogeneous increased enhancement during arterial phase with a fairly rapid washout. On CT scan the mass has brightly enhancing characteristics in arterial phase and early washout with a heterogeneous enhancing pattern on venous phase. She underwent biopsy of the liver lesion and pathology revealed a hepatic perivascular epithelioid cell tumor or PEComa. On H&E staining a haphazard arrangement of epithelioid and spindle cells with cleared-out cytoplasm was seen (Figure 4). The tumor on immunohistochemistry staining was strongly positive for Melan-A (Figure 5) and HMB-45 (Figure 6) and negative for S-100, Hepar-1, ER, PR, desmin, CK7, CK20, CD10, CD117, CD31, synaptophysin, and vimentin.

## 3. Literature Review

Prior to 2011 approximately 100 cases of PEComas originating from different sites and less than 20 cases of Hepatic PEComa were reported. PEComas have a wide variety of presentations and behavior. Reports have suggested that criteria for malignancy include tumor greater than 5 cm, mitotic rate of more than 1 per 50 high power field, and necrosis, but this has not been universally adopted [5]. Uterine PEComas are present in a variety of ways affecting the very young and the very old, with the key factor affecting survival being surgical resectability. The 5-year survival of metastatic uterine PEComa is around 16% [6]. Contrastingly, cutaneous lesions did not recur despite incomplete resection and were seen less commonly [7]. A recent review of renal PEComa has led to prognostic factors such as necrosis, tumor size, and extra renal extension in determining resectability [8].

### 3.1. Pathologic Characteristics of PEComas

Many hypotheses exist regarding the cell of origin and possibilities include neural crest, smooth muscle, or pericytic [9]. Histologically the tumor often appears in a haphazard pattern around a vascular lumen. Cells surrounding the vessels are typically epitheliod and spindle shaped with a clear to pale granular cytoplasm. The tumor is highly vascular with thin-walled vessels that blend with the neoplastic cells [10]. The neoplastic spindle cells have a more granular, eosinophilic cytoplasm. Immunostaining characteristics are consistent with melanocytic and smooth muscle with cells positive for HMB-45/Melanosome, Melan-A, actin, and desmin [11, 12].

### 3.2. Radiologic Characteristics of PEComas

A case series of 32 patients by Tan et al. sought to describe the radiologic characteristics of PEComas. Most PEComas were found to be of low density on CT, hypointense on T1 weighted MRI, and hyperintense on T2. Tumors typically had well-defined borders and enhanced heterogeneously on both arterial and venous phases [13].

### 3.3. Primary Hepatic PEComas

Primary hepatic PEComas appear to be less common than other PEComas. In 2000, 7 young patients with primary hepatic PEComa were reported [14]. In each patient the tumor was located within or abutting the ligamentum teres or falciform ligament. Patients were aged 3–29 and median tumor size was 8 cm. All patients were treated with surgical resection and 5 remained disease free at a median followup of 18 months. One patient developed pulmonary metastases and the other was lost to followup.

Recently, Tan and Xiao published a 7-case series of primary hepatic PEComas [15]. This review focused on the imaging and pathologic characteristics of primary hepatic PEComas. All hepatic PEComas were found to be in livers without a background of cirrhosis or hepatitis. On imaging these lesions were regular, well-defined masses with a homogenous density. They are quickly enhanced in the arterial phase and uniformly become less enhanced on venous and delayed phases of imaging. All primary hepatic PEComas were isolated to the liver without evidence of local or distant metastases, although one patient did have multifocal disease within the liver. PEComas ranged from 2.5 cm to 8.5 cm with a mean of 4 cm [15].

Tan and Xiao also described the pathologic appearance of hepatic PEComas. The gross appearances of the tumors were smooth defined masses with clear boundaries. Microscopically cell membranes were defined as round or polygonal cells with distinct, round medium sized nuclei [15]. Cells were arranged in dense sheets in an epithelial-like pattern with abundant dilated vascularity [16, 17]. Immunohistochemistry of PEComa is characterized by coexpression of melanocytic and muscle markers [16], and hence these tumors are positive for HMB45, Melan-A, S-100, and SMA. IHC was negative for CgA, Syn, CK, CD117, CD10, and CD34.

The natural history of primary hepatic PEComas is quite varied and not yet well established or predictable. Presentation ranged from a palpable abdominal mass [18] to acute abdomen [19]. Treatment in all cases reviewed involved resection of the primary lesion followed by observation. As evidenced by the dilated abundant vasculature on pathology, these tumors can be hemorrhagic which can pose difficulty in resection [20, 21]. Most often there was no recurrence of disease [5, 14, 15, 19, 22–24] after initial resection. One case developed pulmonary metastases [14].

### 3.4. Management of PEComa

The mainstay of treatment of PEComa has been resection. In a large case series of 26 patients by Folpe et al. [14], primary treatment was resection. Only 8 of 24 patients available for followup had recurrence, three as local recurrence and five as metastatic disease. Size (greater than 8 cm) and pathological characteristics (necrosis, high mitotic count) were predictable of increased risk of recurrence [14, 25] with infiltrative margins, high grade nuclear atypia, and vascular invasion used in consideration for designating. If more than 2 high risk features exist, a PEComa was designated as malignant [25].

A recently published review of 234 cases evaluated the benefits of both chemotherapy and radiation therapy for PEComa [26]. They described six cases of neoadjuvant therapy using chemotherapy or chemoradiation with response rates ranging from 0 to 80% with some cases having progression of disease while being on therapy. No response was noted in patients receiving neoadjuvant radiation alone. A total of 19 patients in the review received adjuvant therapy with chemotherapy, radiation, hormonal, or immunotherapy. The majority of adjuvant chemotherapy cases available for followup had recurrent disease within two years. For those patients presenting with metastatic disease, the majority succumbed to their disease with a survival time ranging from 4 to 30 months. Treatment of metastatic disease that occurred after initial resection included further resection, chemotherapy, radiation, imatinib, and rarely mTOR inhibitors, with variable responses [26].

PEComas express p70S6K which is involved in the mTOR pathway [11]. Several reports of metastatic PEComa treated with mTOR inhibitors are described. A 2-year-old girl failed initial resection and chemotherapy had partial response of her liver lesions with sirolimus plus etoposide. A second case of a 63-year-old female who developed metastatic disease 4 months after second resection for a local recurrence was treated with everolimus. Her lung lesions resolved, a retroperitoneal mass significantly reduced in size, and response lasted for approximately 10 months. Both patients were initially treated with imatinib without response and both were living three years after development of metastatic disease [11].

## 4. Conclusion

PEComa is a rare but increasingly recognized tumor. There are characteristic imaging and pathologic findings to confirm the diagnosis. PEComas can display characteristics of both benign and malignant tumors and the primary treatment is resection. Our case adds to the volume of cases, in particular to primary hepatic PEComas, and helps to increase awareness and understanding of this rare tumor.
